# Plug and Pop: A 3D-Printed, Modular Platform for Drug Delivery Using Clinical Ultrasound and Microbubbles

**DOI:** 10.3390/pharmaceutics14112516

**Published:** 2022-11-19

**Authors:** Kushal Joshi, Rajiv Sanwal, Kelsie L. Thu, Scott S. H. Tsai, Warren L. Lee

**Affiliations:** 1 Department of Mechanical and Industrial Engineering, Toronto Metropolitan University, Toronto, ON M5B 2K3, Canada; 2 Institute for Biomedical Engineering, Science and Technology (iBEST), Toronto, ON M5B 1T8, Canada; 3 Keenan Research Centre for Biomedical Science, St. Michael’s Hospital, Unity Health Toronto, Toronto, ON M5B 1T8, Canada; 4 Department of Laboratory Medicine and Pathobiology, University of Toronto, Toronto, ON M5S 1A8, Canada; 5 Biomedical Engineering Graduate Program, Toronto Metropolitan University, Toronto, ON M5B 2K3, Canada; 6 Interdepartmental Division of Critical Care Medicine, University of Toronto, Toronto, ON M5S 1A1, Canada

**Keywords:** ultrasound, microbubbles, drug delivery, gene delivery, 3D-printing, clinical ultrasound transducer

## Abstract

Targeted drug and gene delivery using ultrasound and microbubbles (USMB) has the potential to treat several diseases. In vitro investigation of USMB-mediated delivery is of prime importance prior to in vivo studies because it is cost-efficient and allows for the rapid optimization of experimental parameters. Most in vitro USMB studies are carried out with non-clinical, research-grade ultrasound systems, which are not approved for clinical use and are difficult to replicate by other labs. A standardized, low-cost, and easy-to-use in vitro experimental setup using a clinical ultrasound system would facilitate the eventual translation of the technology to the bedside. In this paper, we report a modular 3D-printed experimental setup using a clinical ultrasound transducer that can be used to study USMB-mediated drug delivery. We demonstrate its utility for optimizing various cargo delivery parameters in the HEK293 cell line, as well as for the CMT167 lung carcinoma cell line, using dextran as a model drug. We found that the proportion of dextran-positive cells increases with increasing mechanical index and ultrasound treatment time and decreases with increasing pulse interval (PI). We also observed that dextran delivery is most efficient for a narrow range of microbubble concentrations.

## 1. Introduction

Targeted drug and gene delivery using ultrasound and microbubbles has emerged as a powerful technique for treating various diseases [1,2,3]. Microbubbles are micron-sized entities containing a high molecular weight gas core encapsulated by a lipid or protein shell. These are frequently used as contrast agents in ultrasound imaging, especially for assessing blood-flow characteristics [4]. Due to the high compressibility of the gas core, microbubbles undergo volumetric oscillations in response to alternating low- and high-pressure cycles of ultrasound. Depending on the ultrasound parameters, the size of the microbubbles, and the composition of the lipid shell, microbubbles can oscillate in two ways, termed stable cavitation and inertial cavitation. Stable cavitation refers to repetitive volumetric oscillations of microbubbles in response to ultrasound, which can continue over many cycles of acoustic pressure [5]. Inertial cavitation refers to the process in which the microbubble diameter usually increases to twice its original size, followed by microbubble collapse and fragmentation into smaller microbubbles [5]. Both stable and inertial cavitation produce a variety of flow patterns in the surrounding fluid. For example, stable cavitation can induce the microstreaming flow in the surrounding fluid, whereas inertial cavitation can lead to the formation of shockwaves and liquid microjets [6,7,8]. If a stable or inertial cavitation of microbubbles occurs in the vicinity of cells, flow patterns generated from these phenomena produce high shear stress on the cell membrane, leading to an increase in membrane permeability, due to the formation of transient membrane pores [6]. This phenomenon is referred to as sonoporation. The mechanical stimulus resulting from high shear stress on the cell membrane also increases the rate of endocytosis [9]. Sonoporation and enhanced endocytosis resulting from ultrasound–microbubble (USMB) exposure can be utilized for the targeted delivery of various therapeutic cargoes, including drugs, plasmids, and siRNA, into cells and tissues [6].

Over the past two decades, USMB-mediated cargo delivery has been studied in vitro using various cell lines and in vivo using various animal models of disease [10,11,12,13]. In vitro investigation of USMB-mediated delivery is of prime importance prior to in vivo studies because it is cost-efficient and allows for the rapid optimization of experimental parameters, such as acoustic pressure, frequency, microbubble concentration/composition, and drug dosage. However, most in vitro USMB delivery studies are carried out with non-clinical, research-grade ultrasound systems [10,14]. Such systems can generate arbitrary ultrasound waveforms with a wide range of frequencies (usually up to 50 MHz). In contrast, clinically approved ultrasound systems are used with a narrower range of parameters. For example, clinical ultrasound systems typically operate at frequencies, less than 12 MHz, and emit waves with conventional sinusoidal waveforms [15]. Since most in vitro studies are carried out with non-clinical ultrasound systems, direct translation of optimized USMB-delivery parameters to clinical settings is challenging.

The main reason for the limited number of studies with clinical ultrasound systems is the lack of an accessible and standardized experimental platform. In this paper, we report a modular 3D-printed experimental setup using a clinical ultrasound transducer that can be used to study USMB-mediated drug delivery in vitro, termed the ultrasound–microbubble cell chamber (UMCC). We demonstrate its utility for optimizing cargo delivery in the HEK293 human embryonic kidney cell line, as well as the CMT167 mouse lung cancer cell line, and we show that cargo delivery varies with ultrasound parameters, including the mechanical index, pulse interval, and exposure time, as well as microbubble concentration.

## 2. Materials and Methods

### 2.1. Engineering UMCC: A 3D-Printed, Modular Platform for USMB-Delivery Testing

**Figure 1:**
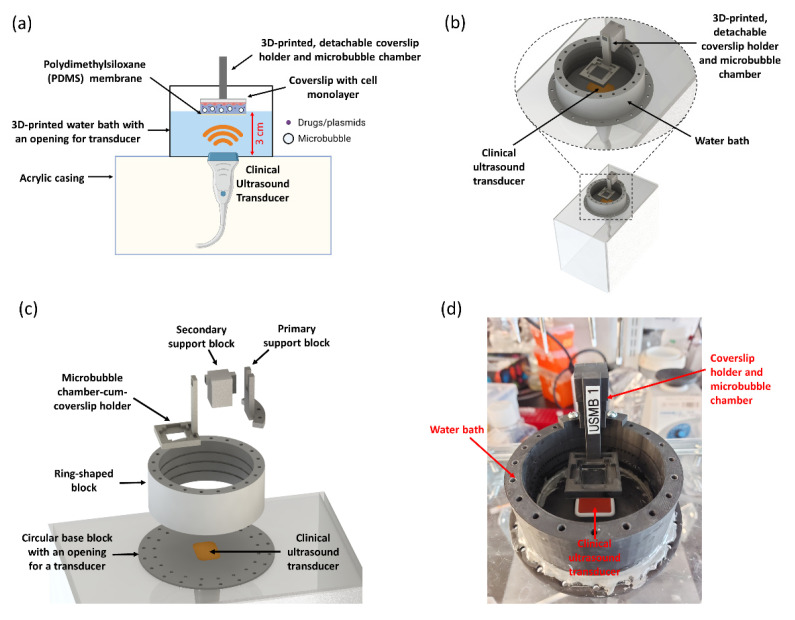
**Schematic and 3D CAD model of UMCC platform.** (**a**) Schematic diagram of UMCC. (**b**) 3D computer-aided design (CAD) model of UMCC. (**c**) Exploded view of UMCC showing different modular parts that are assembled to form the complete setup. Modularity enables easy customization of the platform. (**d**) Actual image of UMCC prototyped using 3D printing and laser cutting.

We used computer-aided design (CAD), 3D-printing, and computer-controlled laser cutting to create the platform (Figure 1). The platform contained a 3D-printed water bath mounted on an acrylic casing. The floor of the water bath had an opening specially designed to fit a clinical ultrasound transducer. In this case, the opening was designed to fit a Phillips S3 phased-array transducer, but the design can be easily modified to fit any other transducer. A 3D-printed, detachable microbubble chamber-cum-coverslip holder was fixed at a focal distance of 3 cm from the face of transducer, based on the maximum intensity (focus) of the ultrasound beam. The base of the microbubble chamber was made using a 0.5 mm thick polydimethylsiloxane (PDMS) membrane (Dow Silicones Corporation, Carrollton, KY, USA), chosen because it has a similar acoustic impedance as that of water. This ensured that the incident ultrasound was completely transmitted through the PDMS membrane into the microbubble solution. The top of the microbubble chamber had specially designed grooves to fit a 22 mm × 22 mm square coverslip (VWR International, Mississauga, ON, Canada) containing a monolayer of cultured cells. The coverslip was inverted and placed in these grooves, such that the cell monolayer was in contact with the solution of microbubbles and drugs. As microbubbles have a natural tendency to float upwards, due to their positive buoyancy, this inverted coverslip configuration allowed microbubbles to accumulate near the cell monolayer and undergo cavitation in close vicinity of the cells.

The entire platform was modular, consisting of individual blocks that can be rapidly assembled. Figure 1c shows the exploded view of UMCC with its individual parts. Each part was designed using the part modelling feature of Solidworks (Dassault Systemes SolidWorks Corp., Waltham, MA, USA), a popular CAD software, and subsequently exported in stereolithography (STL) file format. The STL files were then imported into Eiger 3D printing software (Markforged, Watertown, MA, USA) and printed with Mark Two 3D printer (Markforged, Watertown, MA, USA) using Onyx^TM^, a flagship micro carbon fiber filled nylon composite material from Markforged, Watertown, MA, USA. Following 3D printing, the individual parts of UMCC were assembled. Specifically, the ring-shaped block was mounted on a circular base and fixed using screws to form the water bath. The edges were sealed with aluminum-filled epoxy putty (HY-POXY, Hilton Head Island, SC, USA) to prevent water leakage. The primary support block was fixed to the top of the water bath using screws. The secondary support block had an extrusion which fit tightly into a specially designed groove in the primary support block—analogous to LEGO^®^ blocks, a popular assembly toy. The 3D-printed microbubble chamber-cum-coverslip holder also had a specially designed groove, which fit directly into an extrusion in the secondary support block. Following the assembly of all the 3D-printed blocks, the setup was fixed with the help of screws on an acrylic frame, made using computer-controlled laser cutting. Figure 1d shows the completely assembled UMCC platform. The CAD files (SolidWorks and STL) of all the individual parts of UMCC and detailed assembly schematics will be made freely available upon request.

### 2.2. Clinical Ultrasound System and Transducer

We used a Philips SONOS 5500 clinical ultrasound system and a Philips S3 phased array transducer in all experiments. Experiments were performed in harmonic fusion mode with 1.3 MHz ultrasound transmit frequency and a focal length of 3 cm.

### 2.3. Experimental Setup for Real-Time Visualization of Ultrasound-Mediated Microbubble Cavitation

**Figure 2:**
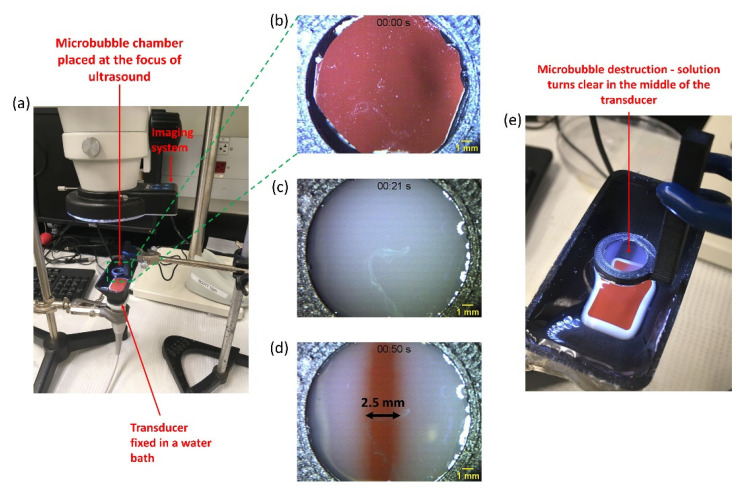
**Real time visualization of microbubble cavitation in response to ultrasound exposure.** (**a**) Image of the 3D-printed setup for real-time visualization of ultrasound-mediated microbubble destruction. It consists of a 3D-printed water bath fixed on Phillips S3 transducer. A small 3D-printed microbubble chamber is placed at the focus of the transducer. The setup is placed under a microscope for real-time imaging. (**b**) Image of the microbubble chamber before addition of bubbles. The transducer face (reddish-orange color) is visible through the chamber. (**c**) Image of microbubble chamber after addition of bubbles. The solution appears milky due to the presence of microbubbles and the transducer face is barely visible. (**d**) Image of the microbubble chamber after application of ultrasound. The solution turns clear in the centre of chamber and the transducer face becomes visible, indicating destruction of microbubbles in the centre of the transducer. (**e**) Image of the setup during microbubble destruction due to ultrasound.

DEFINITY^®^ microbubbles (Lantheus, Billerica, MA, USA) were diluted in phosphate-buffered saline (PBS) (Corning, New York, NY, USA) in a volume ratio of 1:5 and injected into a microbubble chamber placed at the focus of Phillips S3 transducer, as shown in Figure 2a. The entire setup was placed below a microscope for recording real-time videos of microbubble cavitation in response to ultrasound with mechanical index (MI) of 1.6 and pulse interval (PI) of 200 ms.

### 2.4. Transducer Characterization Using Hydrophone Measurements

The ultrasound pressure profile generated by the Philips S3 transducer was measured with a hydrophone. The transducer and hydrophone were immersed in a large water bath and secured in specially designed holders. The hydrophone was placed at a focal distance of 3 cm from the face of the transducer and connected to an oscilloscope and an amplifier. The position of the hydrophone varied along the smaller edge of the transducer, from one end to another, in steps of 1 mm. Ultrasound pressure was measured at each position. All measurements were taken in harmonic fusion mode, with 1.3 MHz ultrasound transmit frequency at MI of 1.0 and PI of 1000 ms.

### 2.5. Cell Culturing

HEK293 (ATCC) and CMT167 (Sigma Aldrich, Oakville, ON, Canada) cells were cultured in Dulbecco’s modified eagle medium (Sigma Aldrich, ON, Canada) supplemented with 10% fetal bovine serum (Sigma Aldrich, ON, Canada) and 1% penicillin–streptomycin solution (Sigma Aldrich, ON, Canada). Cells were cultured on 22 mm × 22 mm square coverslips (VWR International, ON, Canada) in 6-well plates. The coverslips were covered with autoclave tape, leaving only a 4 mm wide region in the center of the coverslip uncovered for cell growth; this ensured that the cells grew only in the area to be sonoporated (based on the hydrophone data). Either 1 million cells (HEK293) or 0.8 million cells (CMT167) were seeded per well and allowed to grow for 24 h at 37 °C in a 5% CO_2_ environment.

### 2.6. Experimental Protocol for Optimizing Delivery of 70 kDa and 4 kDa Dextran

To demonstrate the utility of UMCC for USMB-mediated delivery, we used 70 kDa tetramethylrhodamine dextran (Thermofisher Scientific, ON, Canada) and 4 kDa FITC dextran (Thermofisher Scientific, ON, Canada) as model cargoes. A total of 70 kDa tetramethylrhodamine dextran was added to PBS to yield a final solution of 50 µg/mL dextran in PBS, while 4 kDa FITC dextran was added to PBS to yield a final solution of 100 µg/mL dextran in PBS. Commercial DEFINITY^®^ perflutren lipid microbubbles (Lantheus, MA, USA) were used. DEFINITY^®^ microbubble vials were activated using VIALMIX^®^ shaker (Lantheus, MA, USA). The concentration of activated DEFINITY^®^ microbubbles was measured using a coulter counter (Beckman Coulter, ON, Canada) by adding 5 µL DEFINITY^®^ solution to 20 mL isoton solution (Beckman Coulter, ON, Canada). After measuring the concentration, an appropriate volume of DEFINITY^®^ was added to the dextran-PBS solution to obtain a final microbubble concentration of 8 × 10^7^ microbubbles/mL. The solution was then added to the microbubble chamber, and the coverslip with cultured cells was placed in specially designed grooves on the chamber, such that the cells were in contact with the microbubble solution. The ultrasound was then switched on, and various parameters, namely mechanical index (MI), pulse interval (PI), treatment time, and microbubble concentration, varied. Following ultrasound exposure, the coverslips were incubated at 37 °C for 10 min (HEK293) or 1 min (CMT167). After incubation, coverslips were put in a 6-well plate filled with PBS solution. For all experiments, the following controls were used: ‘Blank’ represents coverslips exposed to PBS alone, ‘Dex’ represents coverslips exposed to a solution of dextran in PBS, and ‘US + Dex’ represents coverslips exposed to a solution of dextran in PBS (without microbubbles), along with ultrasound exposure. ‘MB + Dex’ represents the coverslips exposed to a solution of microbubbles and dextran in PBS in the absence of ultrasound. ‘USMB + Dex’ represents coverslips exposed to a solution of microbubbles and dextran in PBS in the presence of ultrasound.

### 2.7. Quantifying Dextran Delivery by Flow Cytometry

All the experimental groups were analyzed by flow cytometry for quantifying cellular dextran uptake. A total of 500 µL of Trypsin-EDTA solution (Thermofisher Scientific, ON, Canada) was added on the coverslips and incubated at 37 °C for 5 min to allow cells to detach from the coverslip. The cells were then resuspended in 2 mL of Dulbecco’s modified eagle medium (DMEM) (Sigma Aldrich, ON, Canada) supplemented with 10% fetal bovine serum (FBS) (Sigma Aldrich, ON, Canada) and 1% penicillin–streptomycin solution (Sigma Aldrich, ON, Canada). Cell suspensions are centrifuged at 1000 RPM for 3 min, the supernatant decanted, and the cells resuspended in flow cytometry buffer containing 2% BSA (BioShop, ON, Canada) and 2mM EDTA (BioShop, ON, Canada) in PBS. Resuspended cells were, again, centrifuged at 1000 RPM for 3 min, and the pellets were resuspended in 200 µL DAPI staining solution, which is a solution of flow cytometry buffer with DAPI at 1:2000 dilution from 1 mg/mL stock. DAPI staining solution was used for assessing cell viability, as DAPI can only enter and stain the nucleus of cells with compromised cell membranes. Both an unstained control and a live-dead control were used in the analysis. Unstained control represents cells unexposed to dextran or microbubbles and resuspended in plain flow cytometry buffer without DAPI. The live-dead control was prepared from cells cultured in a 6-well plate that were resuspended in DMEM (Sigma Aldrich, ON, Canada) supplemented with 10% FBS (Sigma Aldrich, ON, Canada) and 1% penicillin–streptomycin solution (Sigma Aldrich, ON, Canada). Half of this cell suspension was heated at 75 °C for 8 min, followed by rapid cooling on ice for 4 min to kill the cells by heat shock. This cell suspension was then mixed with the original suspension to make a mixture of live cells and dead cells and, finally, resuspended in DAPI staining solution after centrifugation.

Flow cytometry analysis was performed using a CytoFlex-LX flow cytometer (Beckman Coulter, ON, Canada). For each sample, at least 4000 live cell events were acquired.

### 2.8. Statistics

All the experiments were repeated independently at least 3 times. Data are presented as mean and standard error of the mean. Comparisons between groups were performed using one-way ANOVA testing with post-hoc Tukey’s test (GraphPad Prism, San Diego, CA, USA).

## 3. Results

### 3.1. Characterization of a Clinical Ultrasound Transducer for USMB

Before designing the UMCC, we carried out experiments to visualize microbubble cavitation in real-time in response to ultrasound exposure. The rationale for these experiments was to obtain qualitative information about the spatial distribution of the ultrasound field and how microbubbles respond to it, so that cells could be grown appropriately within the ultrasound field for USMB-mediated delivery. These experiments were performed with the setup described in Section 2.3. Figure 2b shows the microbubble chamber before the addition of microbubbles; the orange-colored transducer face is clearly visible through the transparent PDMS membrane. Figure 2c shows the chamber after the addition of the microbubble chamber. The solution appears milky due to the presence of microbubbles. Figure 2d shows the microbubble chamber after the application of ultrasound with MI of 1.6 and PI of 200 ms. We observed the clearing of the solution starting in the central region of the transducer; this represents the microbubble cavitation. Analysis using ImageJ software showed that the width of this area was approximately 2.5 mm (compared to the 15 mm width of the S3 transducer). This led us to hypothesize that the ultrasound pressure produced by the transducer is highest in this central region of the transducer.

**Figure 3:**
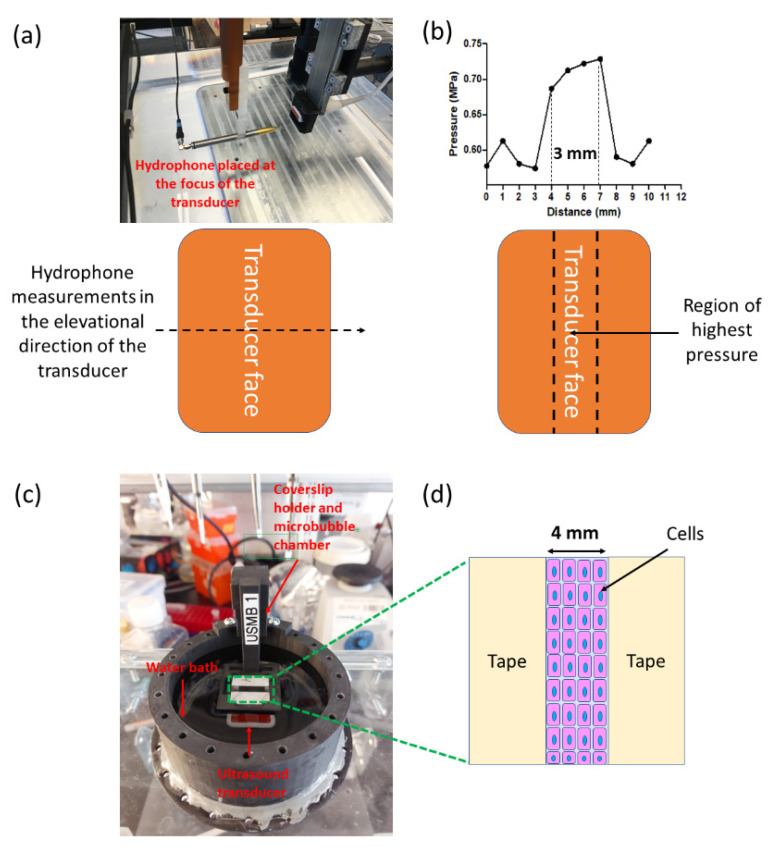
**Characterization of ultrasound transducer using hydrophone.** (**a**) Image of the hydrophone setup used for measuring ultrasound pressure. The hydrophone was placed at a focal distance of 3 cm from the face of the transducer. Hydrophone position was changed in steps of 1 mm to measure the pressure along the elevational direction of the transducer, as indicated by the dotted arrow. (**b**) Ultrasound pressure at the focal plane of the transducer measured using the hydrophone setup. The pressure was highest within a 3 mm wide region at the centre of the transducer. (**c**) Image of UMCC platform with taped coverslip. Cells were cultured within the un-taped, 4 mm wide region, so that all the cells were exposed to highest ultrasound pressure. (**d**) Schematic of the taped coverslip showing cells cultured in the 4 mm wide region.

To confirm this, we next used a hydrophone for characterizing the ultrasound pressure distribution. The hydrophone was placed at a focal distance of 3 cm from the face of the transducer in a large water bath and moved along the smaller edge of the transducer in steps of 1 mm (Figure 3a). Figure 3b shows the ultrasound pressure measured by the hydrophone as a function of its position. The graph indicates that the ultrasound pressure is highest within a 3 mm region in the middle of the transducer, corroborating our hypothesis.

### 3.2. Optimization of 70 kDa Dextran Delivery in HEK293 Cells Using UMCC

**Figure 4:**
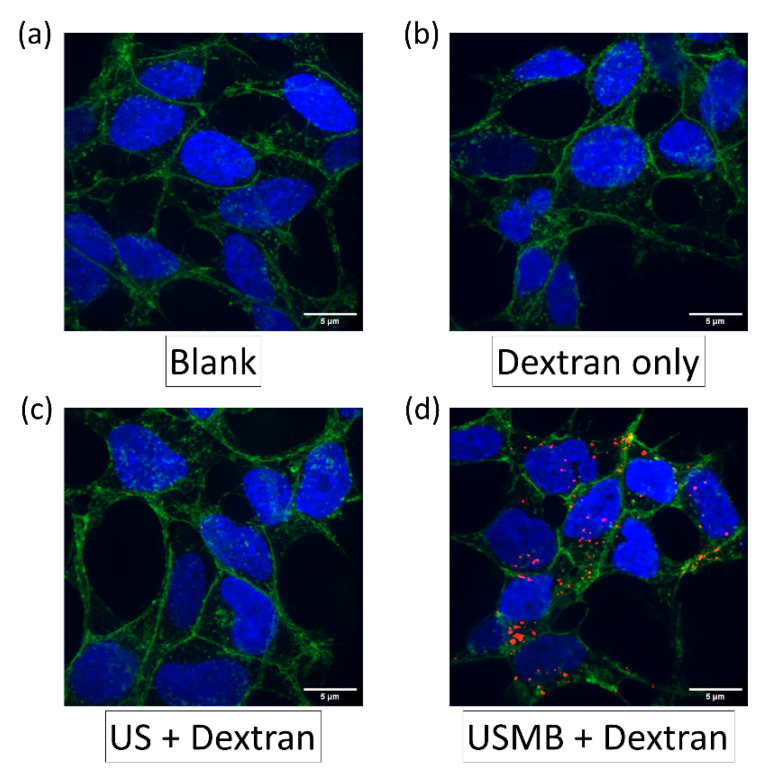
**Representative confocal images of HEK293 cells.** (**a**) Confocal image of ‘Blank’ control group. The ‘Blank’ control represents cells exposed to PBS solution alone. Blue represents ‘DAPI’ used to stain cell nucleus, green represents wheat germ agglutinin tagged with AF-488. (**b**) Confocal image of ‘Dextran only’ control group. The ‘Dextran only’ control represents cells exposed to a solution of 70 kDa dextran in PBS. (**c**) Confocal image of ‘US + Dextran’ control group. The ‘US + Dextran’ control represents cells exposed to a solution of 70 kDa dextran in PBS, along with ultrasound treatment. (**d**) Confocal image of ‘USMB + Dextran’ control group. The ‘USMB + Dextran’ control represents cells exposed to a solution of 70 kDa dextran and microbubbles in PBS, along with ultrasound treatment. Red in the image indicates 70 kDa dextran tagged with tetramethylrhodamine.

Based on these data, we used UMCC to test USMB-mediated cargo delivery using HEK cells. Since the ultrasound pressure generated by the Philips S3 transducer was highest in the center within a 3 mm wide region, we grew cells only in this area by blocking the remainder of the coverslip with autoclave tape (Figure 3d). HEK cells grown in a 4 × 22 mm wide central strip on the coverslip were then utilized for optimizing delivery of 70 kDa tetramethylrhodamine dextran using UMCC. Confocal microscopy images of different experimental groups qualitatively showed a higher uptake in the USMB group, compared to the controls (Figure 4)

**Figure 5:**
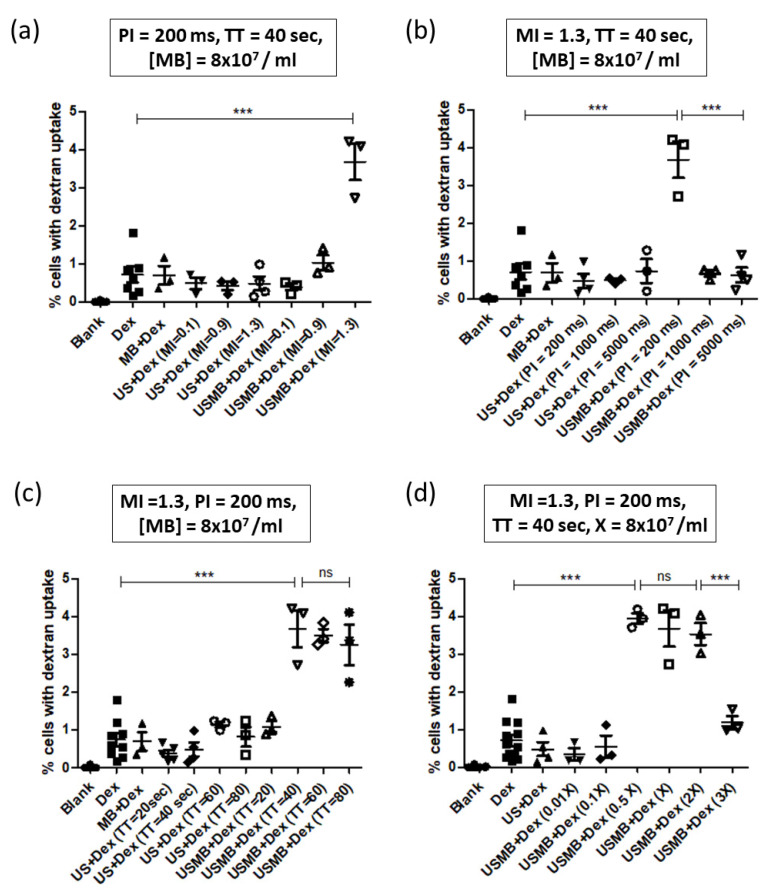
**Optimization of 70 kDa dextran delivery in HEK293 cells by varying different experimental parameters.** (**a**) Plot showing percentage of cells with dextran uptake for different treatment groups as a function of MI. The proportion of dextran positive cells increased significantly at MI of 1.3, compared to the controls (*p* < 0.001). (**b**) Plot showing percentage of cells with dextran uptake for different treatment groups as a function of PI. The proportion of dextran positive cells increased significantly at 200 ms PI, compared to all the other groups (*p* < 0.001). (**c**) Plot showing percentage of cells with dextran uptake as a function of ultrasound treatment time (TT). The proportion of dextran positive cells increased significantly at 40 sec TT (*p* < 0.001). Increasing TT beyond 40 sec did not change in the proportion of dextran positive cells significantly (*p* > 0.05). (**d**) Plot showing percentage of cells with dextran uptake for different treatment groups as a function of the microbubble concentration (X = 8 × 10^7^ bubbles/ml). The proportion of dextran positive cells increased significantly at 4 × 10^7^/ml concentration (*p* < 0.001). Increasing the concentration to 8 × 10^7^/ml and 1.6 × 10^8^/ml did not change in the proportion of dextran positive cells significantly (*p* > 0.05). Further increase in concentration leads to a significant decrease in the proportion of dextran positive cells (*p* < 0.001). In all the experiments, coverslips were incubated for 10 min at 37 °C. One-way ANOVA with post-hoc Tukey’s test was used for assessing statistical significance in all the groups. In all the figures, *** represents *p* < 0.001.

**Figure 6:**
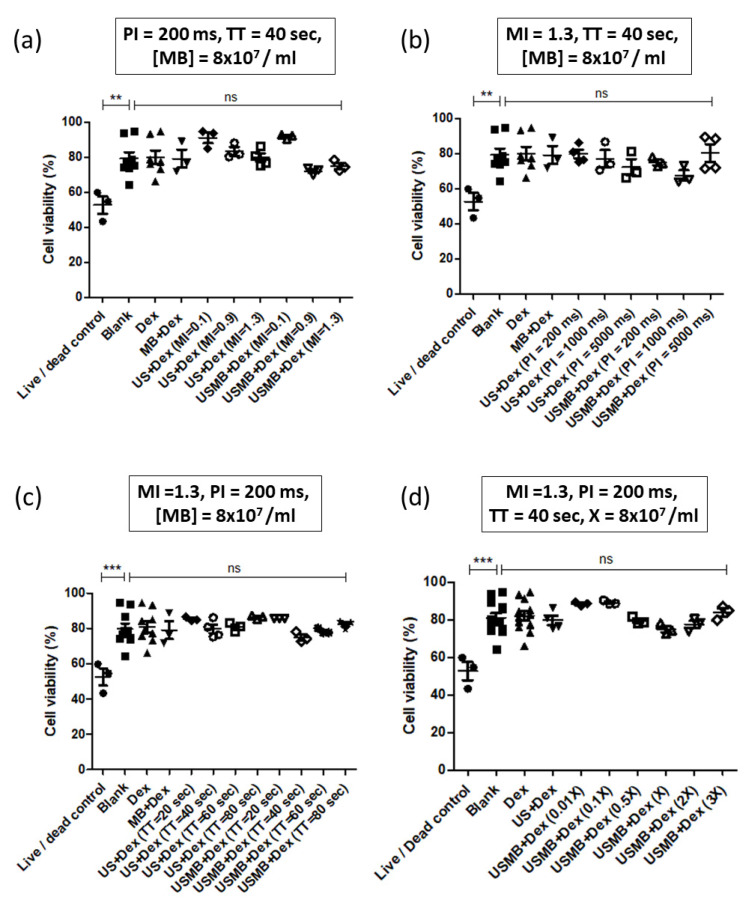
**Cell viability of HEK293 cells under different experimental parameters.** (**a**) Plot showing cell viability of different treatment groups as MI of ultrasound is varied. There was no significant difference in cell viability across all the groups (*p* > 0.05). (**b**) Plot showing cell viability of different treatment groups as the PI of ultrasound was varied. There was no significant difference in cell viability across all the groups (*p* > 0.05). (**c**) Plot showing cell viability of different treatment groups as the ultrasound treatment time (TT) was varied. There was no significant difference in cell viability across all the groups (*p* > 0.05). (**d**) Plot showing cell viability of different treatment groups, as the microbubble concentration was varied in multiples of X = 8 × 10^7^ bubbles/ml. There was no significant difference in cell viability across all the groups (*p* > 0.05). In all the experiments, coverslips were incubated for 10 min at 37 °C. One-way ANOVA with post-hoc Tukey’s test was used for assessing statistical significance in all the groups. In all the figures, ** represents *p* < 0.01 and *** represents *p* < 0.001.

Because confocal imaging of the monolayer may be vulnerable to selection bias (and in order to decrease variability), we decided to quantify dextran uptake by flow cytometry. Figure 5a shows the variation of dextran positive cells with MI of ultrasound at a constant PI of 200 ms and treatment time of 40 s. DEFINITY^®^ microbubble concentration was kept constant at 8 × 10^7^ bubbles/mL, and the dextran concentration was kept constant at 50 µg per ml. The results show that the proportion of dextran positive cells increased with increasing MI, with the highest dextran positive cells obtained at MI of 1.3. As shown in Figure 6a, cell viability was not affected by USMB treatment. However, it should be noted that increasing MI beyond 1.3 led to a significant cell detachment for HEK cells.

We then investigated how PI changes the dextran delivery. Figure 5b shows that, as the PI increased, the proportion of dextran positive cells decreased, with the highest proportion of dextran positive cells obtained at 200 ms PI. Because of the limitations of our clinical ultrasound system, it was not possible to lower the PI below 200 ms. As shown in Figure 6b, cell viability remained constant in all the treatment groups.

We next chose to investigate how dextran delivery changes with ultrasound treatment time. Figure 5c shows that, compared to the controls, the proportion of dextran positive cells in coverslips treated with USMB for 20 s was negligible. However, as the treatment time was doubled, we observed a significant jump in the proportion of dextran positive cells. However, there was no significant increase in the proportion of dextran positive cells at treatment times of 60 or 80 s. In all the treatment groups, cell viability remained unchanged.

Finally, we investigated how varying the concentration of microbubbles affected the dextran uptake by HEK cells. We varied the concentration in multiples of our original concentration (8 × 10^7^ bubbles/mL). Interestingly USMB had no significant effect on dextran uptake at low concentrations of microbubbles (Figure 5d). We observed that the percentage of dextran-positive cells reached a plateau at 4 × 10^7^/mL, with no increase at higher concentrations. In fact, the proportion of dextran positive cells fell to baseline at the highest concentration of bubbles. Cell viability remained constant throughout all the treatment groups (Figure 6d).

### 3.3. Demonstration of 70 kDa and 4 kDa Dextran Delivery in CMT167 Lung Cancer Cells

**Figure 7:**
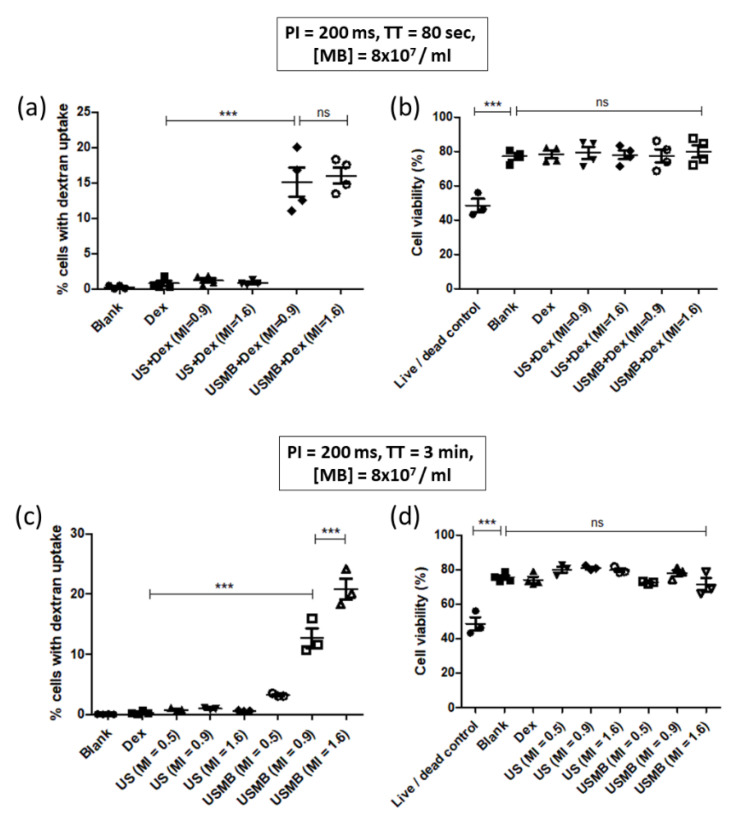
**Variation of 70 kDa and 4 kDa dextran delivery in CMT167 cells with variation in MI.** (**a**) Plot showing percentage of CMT167 cells with 70 kDa dextran uptake for different treatment groups as MI of ultrasound was varied. The proportion of dextran positive cells increased significantly at MI of 0.9, compared to controls (*p* < 0.001). There was no significant increase in the dextran positive cells when MI was increased to 1.6 (*p* > 0.05). (**b**) Plot showing cell viability (%) for different treatment groups shown in panel (**a**). The cell viability did not change significantly in groups treated with USMB, compared to the controls. (*p* > 0.05) (**c**) Plot showing percentage of CMT167 cells with 4 kDa dextran uptake for different treatment groups as MI of ultrasound was varied. The proportion of dextran positive cells increased significantly at MI of 0.9, compared to controls (*p* < 0.001). Increasing the MI to 1.6 led to a significant increase in the proportion of dextran positive cells, compared to MI of 0.9 (*p* < 0.001). (**d**) Plot showing cell viability (%) for different treatment groups shown in panel (**c**). The cell viability did not change significantly in groups treated with USMB, compared to the controls (*p* > 0.05). In all the experiments, coverslips were incubated for 1 min at 37 °C. One-way ANOVA with post-hoc Tukey’s test was used for assessing statistical significance in all the groups. In all the figures, *** represents *p* < 0.001.

UMCC permits the facile comparison of USMB’s effects on different cell types. Given that there is great interest in using USMB to treat tumors, we next tested UMCC on murine-derived CMT167 lung carcinoma cells. CMT167 cells were subjected to USMB-delivery experiments with 70 kDa and 4 kDa dextran to demonstrate the delivery of molecules with different molecular weights. For 70 kDa dextran, we exposed CMT167 cells to ultrasound for 80 s at two different mechanical indices (0.9 and 1.6). We chose this treatment time, as we observed that all the bubbles were destroyed in 80 s for both these mechanical indices. Figure 7a shows that the percentage of dextran positive cells were significantly higher in USMB-treated groups, compared to all the other controls. Moreover, the dextran positive cells did not change significantly when MI was increased from 0.9 to 1.6. Unlike HEK cells, we observed no significant cell detachment at MI 1.6. As shown in Figure 7b, cell viability remained unchanged in all the experimental groups for both dextran molecules.

We next utilized 4 kDa dextran for USMB-mediated delivery to CMT167 cells as a model for smaller molecular weight drugs. In this experiment, we also included a lower MI of 0.5 apart from 0.9 and 1.6. As it takes a significantly longer time (~3 min) for the destruction of microbubbles at MI of 0.5, we increased the treatment time from 80 s to 3 min to ensure that all the bubbles were destroyed at all the MIs. Unlike our experiments with 70 kDa dextran, the proportion of dextran positive cells increased with increasing MI as shown in Figure 7c. The mean proportion of dextran positive cells increased by 8.14% when MI increased from 0.9 to 1.6. Cell viability remained the same in all the experimental groups, as shown in Figure 7d.

## 4. Discussion

There is growing interest in USMB-mediated drug delivery for various diseases, such as ARDS, cancer, and Alzheimer’s disease [1]. Most USMB studies are carried out with non-clinical, research-grade ultrasound systems, which limits the direct clinical translation of in vitro studies. One barrier to clinical translation is the lack of a standardized platform that can be used with clinical ultrasound systems. In this study, we have addressed this issue by developing a simple, low-cost, 3D-printed, and modular platform that can be used in conjunction with a clinical ultrasound system for optimizing USMB-mediated cargo delivery in vitro. Compared to the previously reported platforms, ours is portable and easy to use, as it requires a simple assembly of pre-designed blocks, without the need for additional equipment [16]. Due to its modularity, the platform is versatile, as the design can be rapidly changed to accommodate ultrasound transducers and coverslips of different shapes and sizes. Moreover, the position of the microbubble chamber over the transducer can be changed to investigate the effects of spatial distribution of ultrasound field on the efficacy of drug delivery. Using this platform, we optimized various cargo delivery parameters, such as MI, PI, and ultrasound exposure time, as well as microbubble concentrations, and demonstrated successful cargo delivery with both HEK293 and CMT167 lung carcinoma cells. Similar to previous studies, we utilized dextran as a model drug, as it allows us to vary both the molecular weight and the fluorescent label, facilitating more experiments [17,18]. However, our findings are likely to be applicable to actual drugs, such as antibiotics [1] or chemotherapeutic agents [19]. Some important insights from our dextran optimization studies are listed below.

### 4.1. Proportion of Dextran Positive Cells Increases with Increasing MI

Results with both HEK293 cells and CMT167 lung carcinoma cells show that the proportion of dextran positive cells increases with increasing MI. Provided the ultrasound frequency is kept constant, an increase in MI denotes an increase in acoustic pressure, which directly affects microbubble cavitation. Various investigations on the fundamental physics of ultrasound–microbubble interactions have shown that microbubbles undergo linear oscillations at low acoustic pressures (<50 kPa), while at moderate acoustic pressures (50–200 kPa), they undergo non-linear oscillations. At sufficiently high acoustic pressures (>200 kPa), ultrasound can directly destroy microbubbles, leading to inertial cavitation [20]. Of course, the specific acoustic pressure threshold for linear, non-linear, and inertial cavitation depends on other physical parameters, such as the composition of the encapsulating shell. The increase in the proportion of dextran positive cells with increasing MI might be explained by the changes in the oscillation regimes of microbubbles. Further studies are necessary to confirm this hypothesis.

Our experiments on CMT167 cells also show that the molecular weight of dextran also influences its intracellular delivery, as previously reported by others using different cell lines [17,21,22]. When 70 kDa dextran was used, an increase in MI from 0.9 to 1.6 led to an insignificant change in the proportion of dextran positive cells. However, when 4 kDa dextran was used, the mean proportion of dextran positive cells increased by 8.14% when MI was increased from 0.9 to 1.6. This difference is likely because the lower molecular weight dextran enters the cell more readily than higher molecular weight dextran [21].

### 4.2. Proportion of Dextran Positive Cells Decreases with Increasing PI

As seen from our study on HEK293 cells, the proportion of dextran positive cells decreases with increasing PI (Figure 5b). A lower PI indicates a greater number of pulses in a given amount of time, compared to a higher PI. For example, 40 s treatment with 200 ms PI indicates 200 pulses of ultrasound; however, a 1000 ms PI with the same treatment time indicates just 40 pulses. If the acoustic pressure is greater than the inertial cavitation threshold, a 200 ms PI would destroy a greater number of microbubbles, compared to a 1000 ms PI, due to the greater number of pulses [23,24]. We postulate that this accounts for the decrease in the proportion of dextran positive cells with increasing PI. This is in agreement with previous studies, which have shown that USMB-mediated cargo delivery increases with increasing pulse repetition frequency (i.e., shorter pulse interval) [18,25].

### 4.3. Proportion of Dextran Positive Cells Increases with Ultrasound Exposure Time

As seen from Figure 5c, the proportion of dextran positive cells increases with increasing ultrasound exposure time and ultimately saturates. If the acoustic pressure is sufficiently high to cause inertial cavitation of microbubbles, then an increase in ultrasound exposure time increases the number of microbubbles destroyed per unit of time [26]. When the acoustic pressure is low enough to oscillate microbubbles in linear and/or non-linear regimes, an increase in ultrasound exposure time increases the number of microbubble oscillations per unit of time. In either case, an increase in ultrasound exposure time increases the microbubble cavitation effects, which might explain why the proportion of dextran positive cells increases with ultrasound exposure time [18]. We postulate that the plateau in the proportion of dextran positive cells beyond a certain ultrasound exposure time reflects the depletion of microbubbles left in the solution.

### 4.4. Proportion of Dextran Positive Cells Peaks at a Range of Microbubble Concentrations

One of the most interesting observations from this study was that the proportion of dextran positive cells peaks and then declines as a function of the microbubble concentration (Figure 5d). To the best of our knowledge, this is first time such a result has been reported in the literature. If the microbubble concentration is too low, the proportion of dextran positive cells is negligible. This might be because there is a negligibly low number of microbubbles cavitating in the vicinity of the cells. As we increase the microbubble concentration further, we observe that there is a sudden increase in the proportion of dextran positive cells, compared to the controls, and this proportion stays constant for a range of microbubble concentrations. As we increase the concentration of microbubbles beyond this optimal range, we observe an interesting phenomenon—the proportion of dextran positive cells drops, and there is negligible dextran uptake, compared to the controls. We hypothesize that a very high concentration of microbubbles is ineffective in cargo delivery, due to high ultrasound attenuation by the microbubbles themselves. Ultrasound attenuation is a phenomenon in which the amplitude and intensity of ultrasound wave is reduced as it passes through a certain medium. Previous studies have shown that high microbubble concentrations led to large acoustic attenuation and extreme loss of ultrasound pressure [27]. Moreover, ultrasound imaging studies have shown that high concentrations of microbubbles led to distortion of images, due to large loss in ultrasonic energy [28]. Thus, due to large loss of ultrasound pressure, the microbubble cavitation in the vicinity of cells might not be effective for cargo delivery.

## 5. Conclusions

In summary, we report a novel, 3D-printed, modular platform that is useful for studying USMB-mediated intracellular delivery with a clinical ultrasound transducer. We have demonstrated the utility of this platform for determining the optimal ultrasound and experimental parameters for the successful delivery of dextran of different molecular weights in HEK293 and CMT167 lung carcinoma cells. This platform will allow the facile optimization of USMB-mediated delivery to cells of other cargo, including plasmids and non-coding RNA. Optimization of USMB-enhanced drug and gene delivery is likely to be of clinical utility in diseases such as cancer and acute lung injury [1].

## Data Availability

The CAD files (SolidWorks and STL) of all the individual parts of UMCC and detailed assembly schematics will be made freely available upon request.
